# Comprehensive Fractionation of Antioxidants and GC-MS and ESI-MS Fingerprints of *Celastrus hindsii* Leaves

**DOI:** 10.3390/medicines6020064

**Published:** 2019-06-04

**Authors:** Tran Duc Viet, Tran Dang Xuan, Truong Mai Van, Yusuf Andriana, Ramin Rayee, Hoang-Dung Tran

**Affiliations:** 1 Graduate School for International Development Cooperation (IDEC), Hiroshima University, Hiroshima 739-8529, Japan; viettran1609@gmail.com (T.D.V.); truongmaivan1991@gmail.com (T.M.V.); yusufandriana@yahoo.com (Y.A.); r.rayee12@yahoo.com (R.R.); 2 Faculty of Biotechnology, Nguyen Tat Thanh University, 298A-300A Nguyen Tat Thanh Street, Ward 13, District 4, Ho Chi Minh City 72820, Vietnam; thdung@ntt.edu.vn

**Keywords:** *Celastrus hindsii*, total phenolics, total flavonoids, antioxidant activity, GC-MS, ESI-MS

## Abstract

**Background:** In this study, column chromatography was applied to separate active fractions from the ethyl acetate extract of *Celastrus hindsii*, a medicinal plant widely used in Southern China, Northern Vietnam, Myanmar, and Malaysia. **Methods:** Fourteen fractions from different dilutions of chloroform and methanol were separated by column chromatography and examined for biological activities. **Results:** It was found that a dilution of 50–70% methanol in chloroform yielded the highest total phenolics, flavonoids, and antioxidant activities (1,1-dipheny1-2-picrylhydrazyl (DPPH), 2,2-azinobis (3-ehtylbenzothiazoline-6-sulfonic acid), diammonium salt (ABTS) radical scavenging activity, and *β*-carotene bleaching method measured by lipid peroxidation inhibition). In addition, by gas chromatography-mass spectrometry (GC-MS) and electrospray ionization-mass spectrometry (ESI-MS) analyses, fifteen principal compounds from bioactive fractions belonging to fatty acids, amides, flavonoids, sterols, terpenes, and phenols were identified. Of these compounds, α-amyrin, β-amyrin, hydrazine carboxamide, hexadecanoic acid, fucosterol, (3β)-D:C-friedours-7-en-3-ol, rutin, and 2-hydroxy-1-ethyl ester accounted for maximal quantities, whilst concentrations of other constituents were <5%. **Conclusions:** It is suggested that these identified compounds may greatly contribute to the antioxidant capacity of *C. hindsii* as well as its potential pharmaceutical properties.

## 1. Introduction

*Celastrus hindsii* is a plant species belonging to the Celastraceae family, and is mainly distributed in South America and China [1]. In Vietnam, *C. hindsii* is naturally present in forests and is commonly found in several provinces, such as Ha Nam, Hoa Binh, Quang Ninh, and Ninh Binh. The plants are used as a traditional medicine for treatment of inflammation, and for their anticancer and antitumor properties [2]. Extracts of *C. hindsii* have been used to defend against insects and inhibit pestilences in agriculture. Additionally, it has been known to be a folk medicine for treatment of a plethora of medical ailments, such as stomach disorders, infectious diseases, arthritis, and cancer [3]. Some important compounds, such as maytenfolone A and celasdine B, have been isolated from leaves of *C. hindsii* and have been found to exhibit potent cytotoxicity against cancer cell lines, as well as anti-HIV replication activity [4]. 

For many years, natural products have been effectively used as treatments of various diseases and illnesses. Of them, phenolic constituents are widely studied because they are non-toxic, effective at low concentrations on many biological activities, environmentally friendly, and they can be extracted by low cost technology [5,6]. Besides these benefits, phenolic compounds possess many pharmacological activities, such as antioxidant and anti-inflammatory potential, and show strong inhibition against cardiovascular disease, cancer, and diabetes. Simple phenolic acids and flavonoids are the most common phenolic compounds and generally occur as glycosides and in insoluble forms [7,8]. 

Antioxidants have been used widely in industry and medicine. Their reactive ability can help to prevent aging, cell destruction, and incurable diseases [9]. They have the capability to interrupt radical-chain processes resulting in low activity of free radicals and improve general health, cell rejuvenation, and anticancer mechanisms [10]. Antioxidants also play an important role in delaying or preventing oxidation of oxidizable substrates [11]. 

Due to these beneficial properties of antioxidants, many studies have been carried out to find new natural sources of antioxidants. Research regarding antioxidant extraction from plant tissues have received great attention because of their availability in nature and cost effectiveness. The fortification of exogenous antioxidants in foods, beverages, or medicinal products can be a promising method to counter undesirable effects of oxidative stress [11]. Furthermore, in general, antioxidants also exhibit pharmacological effects, such as antibacterial, antiviral, anti-inflammatory, anti-aging, and anticancer activities [12]. 

Information about antioxidant activity, total phenolic, and flavonoid contents of *C. hindsii* is still limited. This study was carried out to evaluate the antioxidant activity, total phenolic content (TPC), and total flavonoid content (TFC) of *C. hindsii*. The identification of active components was conducted to determine compounds that are responsible for the antioxidant potentials of this plant using (GC-MS) and (EIS-MS) analyses.

## 2. Materials and Methods

### 2.1. Plant Materials

Leaves of *Celastrus hindsii* were collected from Cao Duong commune, Luong Son district, Hoa Binh province, Vietnam, in May of 2017. The dried and sterilized voucher specimens (No. PPBC170506) were deposited. All samples were sterilized, dried in an oven at 30 °C for one week, and then pulverized into a fine powder.

### 2.2. Preparation of Extracts

**Figure 1:**
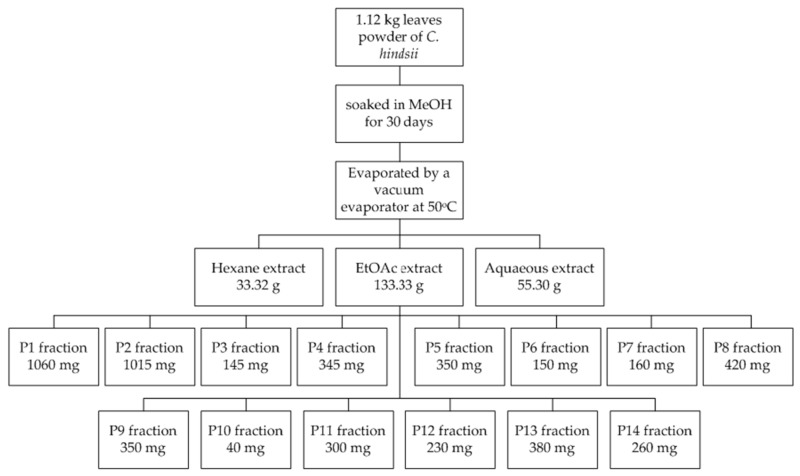
Fractionation of EtOAc extract from powder of *C. hindsii* leaves.

An amount of 1.12 kg powder was soaked in methanol for 30 days in ambient conditions. A rotary evaporator (SB-350-EYELA, Tokyo, Japan) was used to remove the solvent at a temperature of 50 °C. The crude methanol extract was then separated by hexane, ethyl acetate, and an aqueous solution to obtain 33.32 g, 133.33 g, and 55.30 g extracts, respectively. The EtOAc extract, the most active extract in a preliminary test, was fractionated by column chromatography using an eluent gradients technique (chloroform and methanol) to yield 14 fractions (Table 1). These fractions were then subjected to TPC, TFC, and antioxidant assays. The fractionation process of EtOAc extract is described in Figure 1.

### 2.3. Determination of Total Phenolic Contents

The phenolic content was evaluated by the Folin-Cicalteau method [13] with minor modifications. Tested samples were mixed with 0.125 mL of Folin-Ciocalteu reagent and then shaken for 6 min. Then, a volume of 1.25 mL of 7% Na_2_CO_3_ was added. The mixture solutions were adjusted with methanol to a volume of 3 mL, mixed thoroughly, and incubated at ambient temperature in dark conditions. The absorbance was then recorded at 765 nm. Total phenolic content was expressed as milligrams of gallic acid equivalents per gram of extract or fraction (mg GAE/g extract) following a standard curve, which was prepared prior. All samples were analyzed in 3 replicates.

### 2.4. Determination of Total Flavonoid Contents

The total flavonoid content of *C. hindsii* was determined by the aluminum chloride colorimetric method [14]. In this assay, a volume of 100 µL aluminum (III) chloride hexahydrate (2% *w*/*v* in water) was added into 100 µL of sample of rutin standard. After incubation at room temperature and dark conditions for 15 min, the absorbance was measured at 430 nm. Total flavonoid contents were calculated following a standard curve and expressed as mg of rutin equivalent per g extract or fraction (mg RE/g extract).

### 2.5. Antioxidant Properties 

#### 2.5.1. The 2,2-Diphenyl-1-picrylhydrazyl (DPPH) Free Radical Scavenging Activity 

Antioxidant activity of extracts and isolated fractions were estimated by DPPH free radical scavenging method described by [15]. In details, a mixture contained 0.5 mL of each sample, 0.25 mL of 0.5 nM DPPH, and 0.5 mL of 0.1 M acetate buffer (pH 5.5) was prepared and placed in the dark for 30 min at ambient conditions. The absorbance of the reaction was recorded at 517 nm using a microplate reader (MultiskanTM Microplate Spectrophotometer, Thermo Fisher Scientific, Osaka, Japan). Methanol and BHT (10–50 ppm) were used as negative and positive controls, respectively. The antioxidant capacity of the tested samples was calculated using the following equation:

DPPH radical scavenging activity (%) = [(C − S)/C] × 100(1)

where S and C are the corresponding absorbances of reaction with and without sample. The result was expressed as IC_50_ value, which determined the concentration of the sample required to scavenge 50% of DPPH. 

#### 2.5.2. The 2,2′-Azinobis (3-Ethylbenzothiazoline-6-sulfonic acid) (ABTS)

The ABTS method was used to assess the antioxidant property of *C. hindsii* [16]. The ABTS solution was obtained by mixing 7 mM ABTS and 2.45 mM potassium persulfate solution. After incubating the mixture in the dark at room temperature for 16 h, MeOH was added to achieve an absorbance of 0.70 ± 0.05 at 734 nm. A volume of 24 µL of each sample was mixed with 120 µL of ABTS solution, and the mixture was incubated in the dark at ambient conditions for 30 min. The absorbance was read at 734 nm using the microplate reader. BHT (5–125 µg/mL) was chosen as a standard and MeOH was a negative control. The ABTS radical scavenging activity was calculated the same with that of DPPH method.

#### 2.5.3. β-Carotene Bleaching Assay

The β-carotene bleaching method was used to evaluate the antioxidant activity of *C. hindsii* [17]. To begin with, 2.0 mg β-carotene solid and 10 mL chloroform were mixed thoroughly. Afterward, 1 mL of the obtained solution was mixed with 20 µL linoleic acid and 200 mg Tween-40. The chloroform was removed by a vacuum at 45 °C, followed by adding 50 mL oxygenated water to create an emulsion. Next, 0.12 mL of the sample was added into 1 mL of the obtained emulsion in test tubes and the mixtures were incubated at 50 °C. Absorbance was recorded at 492 nm. The reactions were measured every 15 min over 3 h. Methanol and BHT were employed as negative and positive controls. Percentage of lipid peroxidation inhibition (LPI) was determined as follows:

% LPI = B/A × 100(2)

where A and B are absorbance values measured at the start and finish time of the reaction, respectively.

### 2.6. Identification of Chemical Constituents by Gas Chromatography-Mass Spectrometry (GC-MS)

Chemical components of active fractions were identified by using a GC-MS system (JMS-T100 GVC, JEOL Ltd., Tokyo, Japan), according to previous methods [18,19]. The analysis was conducted in DB-5MS column (30 m × 0.25 mm, thickness 0.25 μm) using helium as the carrier gas, performed at a split ratio of 5:1. The injector and detector temperatures were maintained at 300 °C and 320 °C, respectively. The oven temperature was set up as follows: 50 °C without hold time, increasing 10 °C/min to 300 °C, with a 20 min hold. The samples were diluted in MeOH, and injection volume of each sample was 1 µL. The mass range scanned from 29 amu to 800 amu. The identification of identified chemicals was conducted using the mass library of JEOL’s GC-MS Mass Center System Version 2.65 a.

### 2.7. Electrospray Ionization-Mass Spectrometry (ESI-MS) Analysis

The samples were analyzed by ESI-MS in both negative and positive ion mode. The capillary temperature was set at 140 °C (120 °C for S2) and spray voltage of 3.0 KV (2.7 Kv for S2). In the positive mode, the compound analyses were conducted in an ion spray voltage of 3000 V and capillary temperature of 350 °C. The peaks were scanned from 280 to 1000 *m*/*z* [20].

### 2.8. Statistical Analysis 

Statistical analysis was performed by using one-way ANOVA by the Minitab^®^ 16.2.3 (^©^2012 Minitab Inc.; Philadelphia, PA, USA). Turkey’s test was used to identify the significant differences (*p* < 0.05) among the tested samples. The results were expressed as mean values ± standard errors.

## 3. Results and Discussion

### 3.1. Antioxidant Activity, Total Phenolic Content (TPC), and Total Flavonoid Content (TFC) of C. hindsii Extracts

The antioxidant, total phenolic content, and total flavonoid content of extracts are shown in Table 2. The EtOAC extract obtained the maximum amount of TPC and TFC (371.19 and 124.011 mg GE/g extract, respectively). The results showed that TPC, TFC, and antioxidant activity of tested extracts varied. Among the extracts, EtOAc had the highest TPC (371.19 mg GAE/g extract) and TFC (124.77 mg RE/g extract). Similarly, antioxidant activity of this extract was also the strongest (IC50 DPPH and ABTS were 53.38 and 91.08 µg/mL, respectively) compared with other extracts, whilst the hexane extract did not show any antioxidant activity. Due to its strongest antioxidant activity, the EtOAc extract was then separated by column chromatography by using gradient elution technique. 

### 3.2. Total Phenolic (TPC), and Total Flavonoid Content (TFC), and Antioxidant Activity of Fractions Separated from EtOAc Extract

Table 3 shows the TPC, TFC, and antioxidant activity of fourteen fractions separated from EtOAc extract by column chromatography. In general, except for P14, fractions from P9–P13 showed significantly greater TPC and TFC than fractions P1-P8. Of these, the maximum TPC and TPC were observed on the P12-P12 fractions, of which the dilution between chloroform and methanol ranged from 50–70%. When the methanol percentage was either < 30% and > 90%, TPC and TFC were both reduced. The antioxidant capacity of the fractions was accordingly proportional to quantities of TPC and TFC. 

Results in Table 3 also indicate that the dilution between chloroform and methanol strongly influence the antioxidant potential of *C. hindsii,* reflected by both ABTS and DPPH radical scavenging activities, expressed by the IC_50_ values. Of these samples, lower IC_50_ showed stronger antioxidant activity. The fractions P1–P4, P6, and P8 did not show any antioxidant activity, and when the dilution of methanol was 5%, only negligible antioxidant capacity was observed. However, when the dilution of methanol increased to >10%, the ABTS and DPPH radical scavenging activities were rapidly increased. Maximum ABTS and DPPH potentials were found in P12–P13 fractions, where the methanol percentage was increased to 50–70%. However, when the methanol dilution exceeded 70%, the antioxidant capacity was conversely reduced (Table 3). Comparing with the standard BHT, the fractions P12–P13 had the most potential, which might contain constituents active in antioxidant activity, of which the antioxidant levels of individual compounds needed further analysis. Findings of this trial revealed that the dilution of methanol at 50–70% in combination with chloroform provided maximal antioxidant potential in both ABTS and DPPH radical scavenging activities of the medicinal plant *C. hindsii*. In contrast, when methanol accounted for 90% of the solution, both DPPH and ABTS radical scavenging activities were rapidly reduced (Table 3). The antioxidant capacity of *C. hindsii* was also measured by the *β*-carotene bleaching method, as shown in Table 3. Antioxidant activity was expressed by %LPI value against the oxidation of *β*-carotene. Most of the fractions from ethyl acetate extracts had antioxidant activity. The percentage LPI values of EtOAc fractions ranged from 57% to 90% (Table 3). It was observed that all extracts prepared from *C. hindsii* reduced the oxidation of *β*-carotene, although the levels of inhibition varied among fractions. Among isolated fractions, oxidation of linoleic acid was effectively inhibited by P12 fraction (C:M = 1:1; LPI = 90%) followed by P13 (98%) and P9 (87%) fractions. These fractions exhibited antioxidant levels close to that of standard BHT (Table 3). This result showed that *C. hindsii* possessed strong antioxidant capacity.

### 3.3. Correlation Between Phenolic Contents and Antioxidant Activities

**Figure 2:**
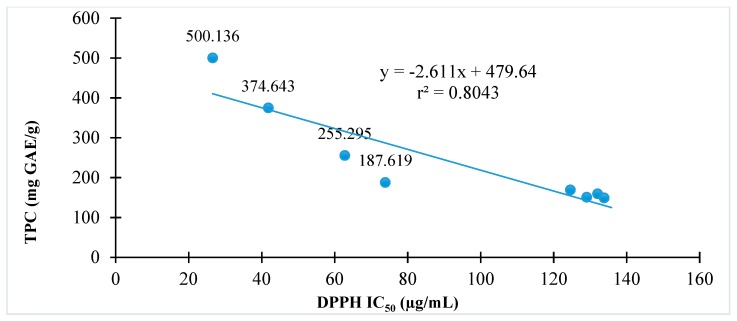
The relationship between antioxidant activity and total phenolic contents of *C. hindsii* by 1,1-diphenyl-2-picryhydrazyl (DPPH) radical scavenging method.

**Figure 3:**
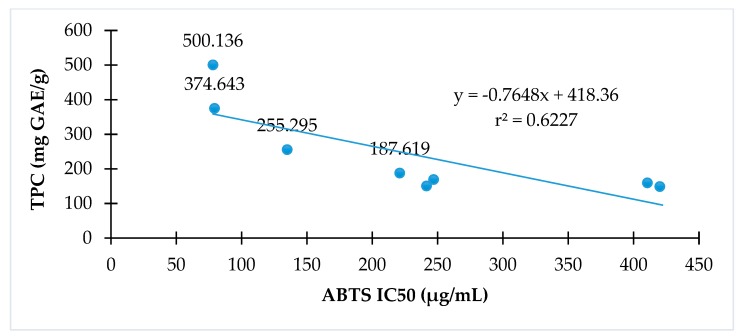
The relationship between antioxidant activities and total phenolic of *C. hindsii* by ABTS radical scavenging activity method.

The relationships of antioxidant activity indicated by either DPPH or ABTS assay to total phenolic contents of *C. hindsii* are presented in Figure 2 and Figure 3, respectively. The results show that total phenolic contents are proportional to DPPH radical scavenging activity (r^2^ = 0.80) or ABTS radical scavenging (r^2^ = 0.62). The fractions with high total phenolic contents have high antioxidant capacity in both DPPH and ABTS assays. 

### 3.4. Identification of Bioactive Compounds by GC-MS and ESI-MS

Bioactive fractions including P1, P4–P14 were analyzed by GC-MS and EIS-MS to reveal the presence of principal compounds, including hexadecanoic acid, α-amyrin, β-amyrin, hydrazine carboxaminde, (3β)-D:C-friedours-7-en-3-ol, fucosterol, β-sitosterol, phytol, dihydroxylacetone, rutin, glycerin, 2′-hydroxyacetophenone, and 2-hydroxy-1-ethyl ester (Table 4). The peaks area (%) were used to compare the concentrations of compounds detected in each fraction. It was found that the presence and concentration of the identified constituents were varied among fractions P1 and P4–P14. Of these fractions, both α-amyrin and β-amyrin showed the maximum concentrations in P1 and P4 (25.56–57.67%). The β-amyrin in P5 and P7 accounted for greater quantity than α-amyrin, however, α-amyrin accounted for 31.74% in P8, whilst no trace of β-amyrin was observed in this fraction. However, both α-amyrin and β-amyrin were not detected in fractions P9–P14 (Table 4). With the exception of P1, the compound hydrazine carboxamide was found in all fractions P4–14. Fraction P4 showed the maximal concentration (38.64%), followed by P13 (21.43%), while other fractions showed lower quantity (1.84–13.75%) (Table 4). Hexadecenoic acid was identified in only P1, P10, and P11, of which P10 showed greater quantity (13.09%). The other principal compounds included fucosterol (43.62%, P5), (3β)-D:C-friedours-7-en-3-ol (29.3%, P5), rutin (7.45%, 12.46%, and 7.43% in P9, P10, and P13, respectively), and 2-hydroxy-1-ethyl ester (20.22%, P13) (Table 4). The other identified chemicals accounted for much lower quantities (<5%). 

## 4. Discussion

Phenolic compounds are significant plant constituents that possess scavenging abilities on free radicals due to their hydroxyl groups. Thus, phenols of plants may contribute directly to their antioxidant action [20]. Interestingly, phenolics and flavonoids are significant compounds in the human diet. These compounds are considered to reduce risk of metabolic syndromes and related complications of type 2 diabetes, and potentially yield many other benefits for human health [21,22,23]. Phenolic and flavonoid compounds possess antioxidant and anti-cancer activities, as well as many other valuable biological potentials [24]. Flavonoids are the largest group of naturally occurring phenolic compounds. They are evaluated to have many biological activities, including antimicrobial, mitochondrial adhesion inhibition, antiulcer, antiarthritic, antiangiogenic, anticancer, and protein kinase inhibitory activities [25]. In addition to these properties, flavonoid compounds are also antioxidants and provide protection against cardiovascular disease, cancer, and age-related degeneration of cell components. In particular, flavonoids have been shown to inhibit tumor development in experimental animal models [26] and possess pharmacological effects, such as ability to inhibit the release of histamines, the adhesion of blood platelets, and the action of lens aldose reductase, to block the inflammatory effects of hepatotoxins, and to act as a heart-stimulant [27]. Phenolics and flavonoids are antioxidants that provide protection against various diseases and can defend against morbidity and mortality from degenerative disorders [8]. 

According to previous studies, *C. hindsii* has exhibited anti-cancer and anti-inflammatory activities [2,3,4]. However, this is the first study to describe the TPC and TFC potential of *C. hindsii*. The high total phenolic and total flavonoid contents of *C. hindsii* plants might contribute important roles in their potent biological activities.

The determination of antioxidant activity is important in estimating the medicinal and pharmaceutical potentials of a plant. DPPH and ABTS radical scavenging activities are among the principal methods used to evaluate antioxidant capacities [11,24]. At room temperature, the free radical DPPH is stable and produces a violet colored solution in methanol, while in the presence of an antioxidant the methanol solution is colorless. DPPH, therefore, is an easy and accurate way to determine the antioxidant activity of plant samples [28]. In biochemistry, 2,2′-azino-bis (3-ethylbenzothiazoline-6-sulphonic acid) or ABTS is usually used in the food industry to determine the antioxidant potential of foods [29]. Accordingly, ABTS is converted to its radical cation by addition of sodium persulfate. This radical cation is blue in color and absorbs light at 734 nm [30]. The ABTS radical cation is reactive towards most antioxidants, including phenolics, thiols, and vitamin C [31]. The antioxidant potential of plant extracts or food products has been extensively measured by the ABTS assay [32]. In addition, β-carotene undergoes rapid discoloration in the absence of an antioxidant because the coupled oxidation of β-carotene and linolelic acid generates free radicals. The linoleic acid free radical formed upon the abstraction of a hydrogen atom in its diallylic methyl groups attacks the highly unsaturated β-carotene molecules. The presence of different antioxidants can hinder the β-carotene bleaching by neutralizing the linoleic free radical and other free radicals formed in the system [33]. 

Because of advantages in DPPH, ABTS, and β-carotene assays, these methods were used to evaluate the antioxidant abilities of *C. hindsii.* It was found that the DPPH and ABTS radical scavenging activities were the strongest in the ethyl acetate fraction, followed by the aqueous solution, whilst hexane showed no activity (Table 2). Similarly, the ethyl acetate fraction extract showed maximal TPC and TFC activities, whilst hexane possessed the lowest (Table 2). Thus, it was observed that the TPC and TFC were proportional to the antioxidant capacity of *C. hindsii*. In addition, results in Figure 1 and Figure 2 and Table 3 show that the DPPH and ABTS radical scavenging activities correspond to the dilution between chloroform and methanol. Of these samples, the percentage of methanol at 50–70% achieved the highest antioxidant capacity. Intensive investigations of *C. hindsii* have reported the presence of many bioactive compounds, including rutin, kaempferol 3-rutinoside, rosmarinic acid, lithospermic acid, lithospermic acid B [34], rosmarinic acid oligomers, celahin B, C, and D [34,35,36], glucosyringic acid, lup-20(29)-ene-3β,11β-diol, lup-20(29)-ene-3-one, and lup-5,20(29)-diene-3-one [37]. In this study, using GC-MS and ESI-MS, 15 major compounds belonging to fatty acid, amide, flavonoid, sterol, terpene, and phenol groups were identified. Of them, α-amyrin, β-amyrin, hydrazine carboxamide, hexadecanoic acid, fucosterol, (3β)-D:C-friedours-7-en-3-ol, rutin, and 2-hydroxy-1-ethyl ester accounted for maximal quantities, whilst concentrations of other constituents were <5%. The fraction P12 exhibited the strongest antioxidant activity against DPPH, ABTS, and β-carotene. The dominant compounds of this fraction revealed by GC-MS and ESI-MS analyses were rutin (12.46%)**,** 2-palmitoylglycerol (6.09%), and 2’-hydroxyacetophenone (4.01%). The presence of these compounds may cause antiradical scavenging activity of this fraction and *C. hindsii*. In literature, the compound rutin, one of the most abundant flavonoids, has been studied to be a potential antioxidant of *C. hindsii* [38,39]. Additionally, this compound has been reported to be effective in treating allergic reactions [39], inflammatation, vasoactivity, tumor growth, bacterial and viral infections, and protozoal contaminations [40]. These pharmacological activities of rutin are mainly attributed to its antioxidant property, particularly as a free radical scavenger [41,42,43]. Moreover, rutin is a type of flavonoid glycoside, known as vitamin P, and has antiplatelet, antiviral, and antihypertensive properties, and has been shown to strengthen the capillaries of blood vessels. These health benefits might be attributed to its high radical scavenging activity and antioxidant capacity [44]. These properties are potentially beneficial in preventing diseases and protecting the stability of the genome. The antioxidant property of rutin was also observed in inhibiting low-density lipoprotein (LDL) peroxidation and Fenton reaction [45]. The compounds α- and β-amyrin are triterpenes of natural origin, commonly detected in various plant materials, such as steam bark residues of *Byrsonima crassifolia* (9 g/kg) [46] and leaves of *Byrsonima fagifolia* (2.3 g/Kg) [47,48,49]. Therapeutic effects of these substances have been confirmed by subsequent in vitro and in vivo assays for various diseases, such as inflammation, microbial, fungal, and viral infections, and cancer cells [50]. In the literature, hydrazine carboxamide was documented as an antimicrobial agent that inhibits the growth of some bacterial strains, such as *Staphylococcus aureus, Klebsiella pneumoniae, Escherichia coli, Pseudomonas aeruginosa,* and some fungal species, such as *Aspergillus niger, Aspergillus flavus, Penicillium citrinum, Candida albicans,* and *Monascus purpereus* [49]. Recently, this compound was found to greatly contribute to arresting the growth of lung cancer cells of *Eclipta alba* [50]. Fucosterol belongs to phytosterol identified in seaweed brown algae, which has been shown to be active as an antidiabetic, antiosteoporotic, and antioxidant [51,52]. 

Findings of this study suggest that *C. hindsii* contains many bioactive compounds that can be exploited for medicinal and pharmaceutical purposes. By in vitro assays, it was found that this plant possesses potent antioxidant properties, which might be attributed to its high total phenolic and flavonoid contents. However, further studies on isolation and purification of these principal constituents from extracts of *C. hindsii* should be carried out to confirm these results.

## 5. Conclusions

This study observed that ethyl acetate was the most effective extracting solvent to extract potential antioxidants from *C. hindsii*. The use of methanol at 50–70% in combination with chloroform provided maximal DPPH and ABTS radical scavenging activity. The GC-MS and ESI-MS analyses revealed the presence of fifteen compounds, with the principal consitutents being α-amyrin, β-amyrin, hydrazine carboxamide, hexadecanoic acid, fucosterol, (3β)-D:C-friedours-7-en-3-ol, rutin, and 2-hydroxy-1-ethyl ester. In order to confirm and expand on these results, further investigation on medicinal and pharmaceutical properties of this plant should be investigated and the isolation and purification of bioactive chemicals from this plant should be elaborated.

## Figures and Tables

**Table 1:** Yields of fractions separated by column chromatography from different elution of chloroform and methanol.

No.	Fraction Codes	Fractions	Quantity (mg)
1	P1	(C:M = 99:1) F_1–20_	1060
2	P2	(C:M = 99:1) F_20–40_	1015
3	P3	(C:M = 99:1) F_40–60_	145
4	P4	(C:M = 99:1) F_60–80_	345
5	P5	(C:M = 99:1) F_80–100_	350
6	P6	(C:M = 99:1) F_100–120_	150
7	P7	(C:M = 97:3) F_1–20_	160
8	P8	(C:M = 95:5) F_1–20_	420
9	P9	(C:M = 9:1) F_1–20_	350
10	P10	(C:M = 8:2) F_1–20_	40
11	P11	(C:M = 7:3) F_1–20_	300
12	P12	(C:M = 5:5) F_1–20_	230
13	P13	(C:M = 3:7) F_1–20_	380
14	P14	(C:M = 1:9) F_1–20_	260

Note: C = chloroform; M = methanol; F_1-120_ = Flask solution 100 mL from 1–120.

**Table 2:** Antioxidant activities, total phenolic content, and total flavonoid content of *C. hindsii* extracts

Extracts	Antioxidant Activity		TPC	TFC
	IC_50_ DPPH (µg/mL)	IC_50_ ABTS (µg/mL)	(mg GAE/g Extract)	(mg GE/g Extract)
Aqueous Extract	108.22 ± 0.48 ^a^	236.62 ± 6.67 ^a^	167.38 ± 0.55 ^c^	112.31 ± 0.16 ^c^
Ethyl Acetate	53.38 ± 0.98 ^b^	91.08 ± 1.01 ^b^	371.19 ± 0.38 ^b^	124.77 ± 0.11 ^b^
Hexane	-	-	2.381 ± 0.89 ^d^	8.73 ± 0.33 ^d^
BHT	7.22 ± 0.89 ^c^	43.40 ± 3.52 ^d^	-	-

The data represent the means ± SE (n = 3); ^a–d^: Similar letters in a column indicated non-significantly different (*p* < 0.05); - = Not detected; TPC = total phenolic content; TFC = total flavonoid content.

**Table 3:** TPC, TFC, and antioxidant capacity of fractions separated from EtOAc extract of *C. hindsii* leaves.

Fractions	Dilutions	TPC (mg GAE/ g Fraction)	TFC (mg RE/ g Fraction)	Antioxidant Activity		
				ABTS IC_50_ (µg/mL)	DPPH IC_50_ (µg/mL)	LPI (%)
P1	(C:M = 99:1) F_1–20_	19.76 ± 0.714 ^k^	40.06 ± 0.16 ^g^	-	-	58.88
P2	(C:M = 99:1) F_20–40_	15.36 ± 0.55 ^l^	29.08 ± 0.28 ^h^	-	-	58.01
P3	(C:M = 99:1) F_40–60_	111.19 ± 0.36 ^h^	41.65 ± 0.16 ^g^	-	-	59.84
P4	(C:M = 99:1) F_60–80_	84.52 ± 1.35 ^i^	41.11 ± 0.06 ^g^	-	-	57.74
P5	(C:M = 99:1) F_80–100_	168.93 ± 0.55 ^e^	93.36 ± 0.67 ^e^	124.57 ± 4.37 ^a^	241.53 ± 6.52 ^b c^	84.25
P6	(C:M = 99:1) F_100–120_	21.31 ± 0.21 ^k^	39.43 ± 0.16 ^g^	-	-	59.32
P7	(C:M = 97:3) F_1–20_	159.28 ± 1.09 ^f^	90.96 ± 0.11 ^e^	132.04 ± 3.43 ^a^	410.60 ± 21.70 ^a^	82.41
P8	(C:M = 95:5) F_1–20_	23.93 ± 0.74 ^j^	40.24 ± 0.21 ^g^	-	-	57.22
P9	(C:M = 9:1) F_1–20_	255.29 ± 0.55 ^c^	120.20 ± 0.16 ^c^	62.78 ± 15.55 ^c^	134.88 ± 2.56 ^d^	87.40
P10	(C:M = 8:2) F_1–20_	187.62 ± 0.95 ^d^	112.31 ± 0.16 ^d^	73.84 ± 5.22 ^b^	221.06 ± 11.86 ^c^	86.09
P11	(C:M = 7:3) F_1–20_	150.50 ± 0.00 ^g^	91.05 ± 0.37 ^e^	129.05 ± 3.09 ^a^	246.98 ± 11.20 ^b^	83.46
P12	(C:M = 5:5) F_1–20_	500.13 ± 0.55^a^	138.17 ± 0.85 ^a^	26.57 ± 0.74 ^e^	78.08 ± 0.66 ^e^	89.76
P13	(C:M = 3:7) F_1–20_	374.64 ± 0.55 ^b^	127.43 ± 3.55 ^b^	41.83 ± 5.3 ^ed^	79.29 ± 1.06 ^e^	88.71
P14	(C:M = 1:9) F_1–20_	148.93 ± 0.55 ^g^	49.61 ± 0.76 ^f^	133.84 ± 1.46 ^a^	420.10 ± 22.50 ^a^	81.89
BHT	Standard	n.d.	n.d.	7.22 ± 0.89 ^f^	43.40 ± 3.52 ^f^	91.86
MeOH	-	n.d.	n.d	n.d	n.d	9.45

Data presented means ± standard deviations (SD). Different letter in a column indicated significantly different by Tukey’s test (*p* < 0.05); - = not detected; MeOH = methanol; BHT = dibutyl hydroxytoluene; positive control of antioxidant assay; TPC = total phenolic contents; TFC = total flavonoid contents; LPI = lipid peroxidation inhibition; GAE = gallic acid equivalent; RE = rutin equivalent; ABTS = 2,2-azinobis (3-ethylbenzothiazoline 6-sulfonic acid); DPPH = 2,2-diphenyl-1-picrylhydrazyl.

**Table 4:** Principal compounds identified in *C. hindsii* by GC-MS and ESI-MS.

Fractions	Retention time (min)	Area (%)	Compounds	Formula	Molecular Weight (g/mol)	Chemical Class
P1	16.75	0.14	Hexadecanoic acid	C_17_H_34_O_2_	270.4507	Fatty acid
	29.04	38.38	β-Amyrin	C_30_H_50_O	426.729	Triterpene
	29.68	57.67	α-Amyrin	C_30_H_50_O	426.729	Triterpene
P4	21.28	38.64	Hydrazine carboxamide	CH_5_N_3_O	75.071	Amide
	29.03	25.56	β-Amyrin	C_30_H_50_O	426.729	Triterpene
	29.64	32.71	α-Amyrin	C_30_H_50_O	426.729	Triterpene
	2.68	1.84	Hydrazine carboxamide	C_6_H_8_O_3_	128.13	Amide
P5	22.1	4.78	β-Amyrin	C_30_H_50_O	426.73	Triterpene
	21.28	29.30	(3β)-D:C-friedours-7-en-3-ol	C_30_H_50_O	426.73	Triterpene
	21.45	43.62	Fucosterol	C_29_H_48_O	412.70	Sterol
	29.62	13.00	α-Amyrin	C_30_H_50_O	426.73	Triterpene
P6	2.68	13.75	Hydrazine carboxamide	CH_5_N_3_O	75.071	Amide
	29.01	16.40	β-Amyrin	C_30_H_50_O	426.729	Triterpene
	29.81	1.10	α-Amyrin	C_30_H_50_O	426.729	Triterpene
P7	2.68	9.36	Hydrazine carboxamide	CH_5_N_3_O	75.07	Amide
	16.75	0.14	Hexadecanoic acid	C_17_H_34_O_2_	270.45	Fatty acid
	28.44	6.64	β-Sitosterol	C_29_H_50_O	414.71	Sterol
	29.59	1.85	β-Amyrin	C_29_H_50_O	426.72	Triterpene
P8	2.64	8.32	Hydrazine carboxamide	CH_5_N_3_O	75.071	Amide
	15.85	3.53	Phytol	C_20_H_40_O	296.53	Diterpene
	29.6	31.74	α-Amyrin	C_30_H_50_O	426.72	Triterpene
P9	2.68	7.52	Hydrazine carboxamide	CH_5_N_3_O	75.071	Amide
	3.61	4.33	Dihydroxyacetone	C_3_H_6_O_3_	90.078	Glycerone
	19.74	7.45	Rutin	C_27_H_30_O_16_	610.52	Flavonoid
P10	2.64	11.05	Hydrazine carboxamide	CH_5_N_3_O	75.071	Amide
	16.75	13.09	Hexadecanoic acid	C_17_H_34_O_2_	270.45	Fatty acid
P11	2.64	13.54	Hydrazine carboxamide	CH_5_N_3_O	75.071	Amide
	16.75	5.14	Hexadecanoic acid	C_17_H_34_O_2_	270.45	Fatty acid
P12	2.68	12.21	Hydrazine carboxamide	CH_5_N_3_O	75.07	Amide
	4.66	3.43	Glycerin	C_3_H_8_O_3_	92.09	Glycerin
	11.19	4.01	2’-Hydroxyacetophenone	C_8_H_8_O_2_	136.15	Phenol
	19.74	12.46	Rutin	C_27_H_30_O_16_	610.52	Flavonoid
	21.92	6.09	2-Hydroxy-1-ethyl ester	C_19_H_38_O_4_	330.509	Phenol
P13	2.69	21.43	Hydrazine carboxamide	CH_5_N_3_O	75.071	Amide
	19.74	7.43	Rutin	C_27_H_30_O_16_	610.52	Flavonoid
	21.92	20.22	2-Hydroxy-1-ethyl ester	C_19_H_38_O_4_	330.50	Phenolic
P14	2.64	4.04	Hydrazine carboxamide	CH_5_N_3_OCH_5_N_3_O	75.071	Amide
